# 

**Published:** 1874-09

**Authors:** 


### Table of Contents.

### ORIGINAL COMMUNICATIONS.

Normal Ovariotomy.—*By Prof. J. T. Gilmore, M.D., Mobile, Ala.* ....... 321
Report of Twenty-Seven Operations for Cataract.—*By A. W. Calhoun, M.D., Atlanta, Ga.* ....... 330
Torsion of Arteries.—*By Harry L. Sims, M.D., New York* ....... 337
Hemorrhagic Malarial Fever.—*By Wm J. Johnson, M.D., Fort Gaines, Ga.* ....... 340
Normal Ovariotomy—Menstrual Physiology.—*By Ch. Rauschenberg, M.D., Atlanta, Ga.* ....... 350
Phytolacca in Mastitis.—*By L. Alexander, M.D., Yorkville, S. C.* ....... 352
Acute Glossitis.—*By James O. Lewis, M.D., Greenwood, Fla.* ....... 353
Congenital Malformations.—*By M. D. Mooney, M.D., Taylor's Creek, Ga.* ....... 354

### OUR EXCHANGES.

London Doctors ....... 355
Functional Derangements of the Liver ....... 358
Recreative Change ....... 362
Resuscitation from Chloroform ....... 363
Removal of Both Ovaries—Effect ....... 364
Carbuncle, Local Treatment .......
Dr. Sage's Catarrh Remedy ....... 365
Transfusion from Lamb's Blood .......
Silicate of Soda in Gonorrhœa .......
Novel Method of Extracting Teeth .......
Umbilical Hernia in Infants ....... 366
Law of Missouri concerning the Practice of Medicine .......
Euthanasia ....... 367
Golden Farnia Cologne .......
New Application for Old Ulcers .......
Drainage in Ovariotomy ....... 368
Death following use of Sponge Tents ....... 369
Division Tendo-Achilis—Silver suture ....... 371
Phytolacca for Cockroaches ....... 373
Hereditary character of Cancer .......
New treatment of burns ....... 374
What next? ....... 375

### BIBLIOGRAPHICAL.

Parrish's Practical Pharmacy ....... 376
Charles West on Children ....... 377
Flint's Medical Essays .......
Pavy on Food and Dietetics ....... 378
Lincoln's Electro-Therapeutics .......
Hartshorne's Conspectus of Medical Sciences ....... 379
Transactions Medical Association of Georgia .......
Transactions South Carolina Medical Association ....... 380
Transactions Medical and Chirurgical Faculty of Maryland ....... 381
Nomenclature of Diseases, (Woodworth) ....... 382

### EDITORIAL.

Editorial Notes ....... 382
Acknowledgments ....... 383

